# Assessing the Association between Leptin and Bone Mineral Density in HIV-Infected Men

**DOI:** 10.1155/2012/103072

**Published:** 2012-08-27

**Authors:** Madhu N. Rao, Morris Schambelan, Viva W. Tai, Donald I. Abrams, Hootan Khatami, Peter J. Havel, Giorgos Sakkas, Kathleen Mulligan

**Affiliations:** ^1^Department of Medicine, University of California, San Francisco, CA 94143, USA; ^2^Division of Endocrinology, San Francisco General Hospital, 1001 Potrero Avenue, Building 30 Room 3501K, San Francisco, CA 94110, USA; ^3^Division of Hematology and Oncology, San Francisco General Hospital, 995 Potrero Avenue, San Francisco, CA 94110, USA; ^4^Daiichi Sankyo Pharma Development, 399 Thornall Street N. 45, Edison, NJ 08837, USA; ^5^Department of Molecular Biosciences, School of Veterinary Medicine and Department of Nutrition, University of California, Davis, 1093 Haring Hall, Davis, CA 95616, USA; ^6^Department of Medicine, University General Hospital of Larissa, University of Thessaly, Mezourlo, TK 411-10 Larissa, Greece

## Abstract

HIV-infected individuals are at risk for decreased bone mineral density (BMD). The known risk factors for bone loss do not fully explain the increased risk in this population. There is emerging evidence that leptin, a hormone secreted by adipocytes, plays an important role in bone metabolism. Several studies have assessed the relationship between leptin and bone density in healthy adults, but there are few such studies in HIV-infected individuals. Furthermore, HIV infected individuals on antiretroviral therapy are at increased risk for altered fat distribution, which may impact the relationship between leptin and BMD. In a cross-sectional analysis of data in 107 HIV-infected men, we determined whether serum leptin levels were associated with whole-body BMD and bone mineral content measured by dual-energy X-ray absorptiometry (DEXA), after adjusting for confounders including body fat distribution. We found an inverse association between leptin and bone density in those with peripheral lipoatrophy, defined objectively as <3 kg appendicular fat by DEXA, but no such relationship was seen in those with >3 kg appendicular fat. This result suggests that fat distribution may modify the relationship between leptin and bone density.

## 1. Background

Patients with HIV infection are at risk for decreased bone density [1]. This is due to both HIV-specific factors (i.e., use of tenofovir, starting antiretroviral therapy) and generalized factors that are present in this population (including hypogonadism, smoking, alcohol use, and low body weight). However, these etiologies do not fully explain the increased risk for bone loss that is observed [2–4]. In non HIV-infected populations, there is evidence that leptin, a hormone mainly secreted by adipocytes, plays an important role in bone metabolism [5]. In healthy adults, increased fat mass is associated with increased bone density [5] as well as increased circulating leptin concentrations. This has led to the suggestion that leptin may mediate the positive effects of fat mass on bone density. 

Whereas cross sectional studies in healthy women have consistently shown a positive association between leptin levels and bone mineral density (BMD) [6–8], studies in men as well as in rodent models have shown either no association [6, 7], or an inverse relationship [9, 10]. However, leptin levels may be influenced by the specific distribution of body fat (i.e., central versus peripheral) [11, 12]. In HIV-infected individuals, decreased peripheral fat and central fat accumulation are common due to a combination of HIV infection itself as well as antiretroviral medications (such as stavudine, zidovudine, and, potentially, protease inhibitors) [13]. In this study, we hypothesized that circulating leptin concentrations would be associated with whole body BMD and bone mineral content (BMC) in such patients, after adjusting for altered body fat distribution. This hypothesis was assessed in 107 HIV-infected men from the early HAART era, prior to widespread use of tenofovir, an antiretroviral that has been consistently associated with loss of BMD [14]. 

## 2. Methods

### 2.1. Subjects

We performed a retrospective, cross-sectional analysis of data in 107 HIV-infected men who underwent whole-body dual X-ray absorptiometry (DEXA) scan and fasting blood draws at the Clinical Research Center (CRC) at San Francisco General Hospital (SFGH) between 1998 and 2002. The subjects completed these assessments either during participation in an observational cohort study [15] or at screening or baseline visits for three different intervention studies [16–18]. None of the subjects were receiving any experimental metabolic therapies at the time of data collection. For the reasons stated above, we excluded subjects whose ART regimens included tenofovir.

### 2.2. Body Composition

Total body fat, lean body mass (LBM), BMD, and BMC were determined by whole body DEXA scan (Lunar DPX, Madison WI USA). Manual analyses of appendicular (arm + leg) and trunk fat were performed using soft-tissue and skeletal landmarks [19]. 

### 2.3. Laboratory Assays

Serum testosterone levels were measured by radioimmunoassay (Diagnostic Products, Inc, Los Angeles, CA USA). Fasting serum leptin and insulin concentrations were measured by radioimmunoassay (Linco Research, Inc) as previously described [20]. Blood for these assays were drawn at the time the DEXA scan was done. 

### 2.4. Statistical Analysis

Our primary outcomes were whole body BMD and BMC, and the primary predictor was serum leptin. We examined the distribution of all variables; those with skewed distribution were natural log transformed. Mean + SD or median (interquartile range [IQR]) for skewed variables were calculated. We performed bivariate analysis using scatter plots and linear regression for numerical predictors, and chi-square test for dichotomous predictors. The dichotomous predictors included race (Caucasian/non-Caucasian) and antiretroviral medications (yes/no) as described below. We then performed multivariate analyses to account for confounders. Potential confounding variables were identified a priori and included total body fat, percent body fat, trunk fat, appendicular fat, lean body mass (LBM), percent LBM, testosterone levels, years of HIV infection, CD4 count, and classes of antiretroviral medications. If the covariate was associated with BMD or BMC with a *P*-value <0.05 in univariate analyses, it was included within the multivariate model. Age and race were always included as covariates in the multivariate models. We first performed multivariate analysis in the entire population. We then performed further analyses in subjects with lipoatrophy, using the objective definition of Martínez et al. [21] of <3 kg appendicular fat, compared to those with >3 kg of appendicular fat. All analyses were performed using STATA 10.0 (College Station, TX). To allow for better comparisons, we have reported the standardized regression coefficients. 

## 3. Results

### 3.1. Baseline Characteristics and Univariate Analysis

A total of 107 HIV-infected men were included in the study; 69% were Caucasian, with a mean age of 46 ± 9 years, BMI 25.1 ± 3.6 kg/m^2^, and body fat of 15.0 ± 7.5% (Table 1). One hundred and one subjects (94%) were on antiretroviral therapy at the time they were studied; 100 (93%) were on nucleoside/nucleotide reverse transcriptase inhibitors (NRTIs, including 57 on stavudine); 92 (86%) were on a protease inhibitor (PI), and 21 (20%) were on a non-nucleoside reverse transcriptase inhibitor (NNRTI). The median CD4 count was 377 cells/*μ*L (IQR 243–583) and average duration of HIV infection was 11 ± 5 years. The study population included a wide range of body sizes, as reflected by the range of weight (47–106 kg), BMI (16.9–38.6 kg/m^2^), total body fat (3.1–35.3 kg), trunk fat (1.2–20.8 kg), and appendicular fat (1.5–14.3 kg). The median leptin concentration was 3.2 ng/mL (IQR 2.1–5.7). Leptin and total body fat were moderately correlated (Pearson's correlation coefficient =0.6, *P* < 0.001).

In univariate regression, there was no statistically significant association between age, race, percent body fat, percent LBM, testosterone level, years of HIV infection, CD4 count, PI, NRTI, or NNRTI use and BMD or BMC. On the other hand, LBM and total body fat, as well as appendicular and trunk fat, were significantly positively associated with both BMD and BMC. Serum insulin was positively associated with BMD, but not BMC. 

### 3.2. Leptin and Bone Density in the Entire Cohort

In the group as a whole, we found no statistically significant association between natural log-transformed leptin levels and total BMD or BMC in either bivariate analysis or multivariate analyses (Table 2). To assess for potential nonlinear associations between leptin and bone density, we divided leptin into quartiles, but this also failed to show a statistically significant association (*P*-value for linear trend =0.11 and 0.15 for BMD and BMC, resp.). 

### 3.3. Leptin and Bone Density in Subjects with Low Levels of Appendicular Fat

Thirty-three subjects met the definition of peripheral lipoatrophy (<3 kg of appendicular fat). In this group, we found a robust and statistically significant inverse relationship between circulating leptin concentrations and both BMD and BMC (Table 2). This statistically significant relationship was evident in the bivariate analysis and persisted after adjustment for age, race, LBM, trunk fat, and total fat mass. Specifically, for every one SD increase in ln leptin, BMD decreased by 0.92 SD and BMC by 0.80 SD. A formal assessment of the interaction term leptin × appendicular fat revealed that it was statistically significant (*P* = 0.01). 

In subjects with >3 kg of appendicular fat (*n* = 74), there was no significant relationship between leptin and bone density. Further analysis by tertiles of appendicular fat (i.e., <3 kg, 3–7 kg and >7 kg) revealed no significant association between leptin and bone mineral density in the upper two tertiles (*P* = 0.70 and 0.40, resp.). To determine whether leptin was a surrogate marker for appendicular fat, we assessed the association between appendicular fat and bone density. After adjustment for confounders (age, race, LBM, and truncal fat), there was no statistically significant relationship between appendicular fat and whole body BMD or BMC. 

Addition of serum insulin concentrations to the multivariate analysis did not significantly change any of our results (data not shown), either in the entire cohort (*n* = 107), or in the subgroups (i.e., those with <3 kg of appendicular fat and those with >3 kg of appendicular fat). 

## 4. Discussion

In this analysis of data in a diverse group of 107 HIV-infected men, we found no statistically significant relationship between serum leptin levels and whole-body BMD or BMC. However, in the 33 subjects with <3 kg of appendicular fat, we found a robust inverse association between leptin and bone density that was statistically significant, despite the small number of subjects. Adjusting the model for potential confounders such as age, race, LBM, serum insulin, and total and trunk fat did not significantly change the results. 

Studies in HIV-negative individuals have shown differing relationships between circulating leptin and BMD in men and women. Reports in women have consistently found a positive association between leptin and BMD [6, 7], while those in men have found either no association or an inverse relationship [6, 7, 9]. These inconsistencies led us to speculate that the relationship between leptin and BMD may be affected by fat distribution. We analyzed the data both in our entire cohort, as well as in those with peripheral lipoatrophy as defined by <3 kg of appendicular fat. This definition of peripheral lipoatrophy was based on various studies in which participants with clinically evident lipoatrophy were found to have an average of 3 kg of appendicular fat [21]. In an earlier study of HIV infected individuals, Madeddu et al. observed an inverse relationship between circulating leptin concentrations and whole-body BMD in both men and women [22]. In contrast, in our study of men, we found an inverse relationship only among those with lipoatrophy defined using an objective measure (limb fat by DEXA). The reasons for these divergent results in both men with HIV infection (our study versus that of Madeddu) and seronegative men are not known. The results of the current study raise the possibility that differing relationships between leptin and BMD in men versus women in the general population may be explained by different distribution of fat in the sexes (i.e., central versus peripheral). In other words, fat distribution may modify the relationship between leptin and bone density. Indeed, one study in children found significant differences in circulating leptin concentrations depending on the distribution of body fat [12], and an *in vitro* study found differing levels of leptin mRNA in subcutaneous versus intra-abdominal fat [11]. 

This study has a number of limitations. First, we were able to assess only whole body BMD and BMC and did not have direct regional measurements of BMD in the spine or hip. Second, it is a cross-sectional study and our findings are associative in nature. Furthermore, the data used in this study are from the early HAART era, and although antiretroviral medications were not identified as potential confounders in our analyses, we have limited information regarding the duration of medication exposure. Despite the fact that current antiretroviral regimens differ from those in this study, we believe that our findings provide insight regarding the relationship between leptin and bone density. The strengths of our study include the uniformity of gender and study measurements and the ability to objectively measure body fat distribution using whole body DEXA scans in a population with a wide range of body sizes and total and regional fat content. Lastly, the study was performed prior to the widespread use of tenofovir, thereby eliminating the confounding effects of this medication on bone density.

In summary, this study further extends our knowledge by suggesting a role for not just total fat but also fat distribution in explaining the relationship between serum leptin concentrations and bone density. We found that peripheral fat modified the relationship between bone density and leptin levels in HIV-infected men. Specifically, an inverse association between bone density and leptin levels was observed only in subjects with low amounts of peripheral fat. Further studies are required to confirm our findings and further clarify the relationship between fat distribution, bone density and circulating leptin concentrations. 

## Figures and Tables

**Table 1:** Baseline characteristics^∗^.

Characteristics	*n* = 107 men
Age (years)	46.2 ± 9.2
Race/ethnicity (% Caucasian)	69
Height (cm)	175.9 ± 6.2
Weight (kg)	77.7 ± 11.7
Body mass index (kg/m^2^)	25.1 ± 3.6
Years of HIV infection	10.6 ± 5.0
Antiretroviral treatment (%)	94
PI (%)	86
NRTI (%)	93
NNRTI (%)	20
CD4 count (cells/*μ*L)	377 (243, 583)
Testosterone (ng/dL)	525 ± 220
Leptin (ng/mL)	3.2 (2.1, 5.7)
Insulin (*μ*IU/mL)	17.1 ± 13.3
BMC-total body (grams)	2867 ± 382
BMD-total body (g/cm^2^)	1.184 ± 0.081
Total body fat (kg)	15.0 ± 7.5
Truncal fat (kg)	9.1 ± 4.5
Appendicular fat (kg)	5.1 ± 3.2
Lean body mass (kg)	59.7 ± 7.6

^∗^Mean ± SD (for normally distributed data) or median (IQR) (for skewed data).

**Table 2:** Multivariate regression models.

Primary predictor	Total BMD		Total BMC	
	Std Reg Coeff^#^ (95% CI)	*P*-value	Std Reg Coeff^#^ (95% CI)	*P*-value
All subjects (*n* = 107)				
Leptin (ln)^∗^	0.08 (−0.11, 0.27)	0.38	0.06 (−0.13, +0.25)	0.54
Leptin (ln)^∗∗^	−0.11 (−0.34, +0.12)	0.35	−0.19 (−0.38, +0.01)	0.07
Subjects with lipoatrophy (*n* = 33)				
Leptin (ln)^∗^	−0.68 (−1.22, −0.14)	**0.02**	−0.74 (−1.27, −0.21)	**<0.01**
Leptin (ln)^&^	−0.92 (−1.62, −0.22)	**0.01**	−0.80 (−1.32, −0.29)	**<0.01**

^#^Standardized regression coefficient.

^∗^Adjusted for age and race.

^∗∗^Adjusted for age, race, truncal fat, appendicular fat, and lean body mass.

^&^Adjusted for age, race, lean body mass, truncal fat, and total fat.
